# The predictive value of abnormal uteroplacental and fetal Doppler indices for adverse perinatal outcomes in hypertensive disorders of pregnancy: a systematic review and meta-analysis

**DOI:** 10.3389/fmed.2026.1923180

**Published:** 2026-08-12

**Authors:** Peng Zhou, Zhongfeng Tang, Siyuan Li, Wenbo Zhang, Jiawei Song, Fuhua Gao, Lei Yang

**Affiliations:** 1 Gansu University of Chinese Medicine, Lanzhou, Gansu, China; 2 Prenatal Diagnosis Center, Gansu Provincial Maternity and Child Health Care Hospital, Lanzhou, Gansu, China; 3 Department of Ultrasound Medicine, Gansu Provincial Maternity and Child Health Care Hospital, Lanzhou, Gansu, China

**Keywords:** adverse perinatal outcomes, cerebroplacental ratio, GRADE assessment, hypertensive disorders of pregnancy, meta-analysis, uterine artery pulsatility index, uteroplacental and fetal Doppler ultrasound

## Abstract

**Objective:**

To systematically evaluate the predictive value of abnormal uteroplacental and fetal Doppler indices for adverse perinatal outcomes in patients with hypertensive disorders of pregnancy (HDP), quantify the strength of association, and assess the certainty of evidence to optimize prenatal monitoring protocols.

**Methods:**

Based on PRISMA guidelines, we searched Cochrane CENTRAL, PubMed, Embase, and SCIE for observational cohort studies published between January 2016 and March 2026. Included studies comprised singleton pregnant women with HDP who underwent uteroplacental and fetal Doppler assessment (e.g., UtA-PI, UA, MCA, or CPR) and reported perinatal outcomes. Risk of bias was assessed using the Newcastle-Ottawa Scale (NOS), and the certainty of evidence was graded using the GRADE framework. This study was registered with PROSPERO (CRD420261331863).

**Results:**

Thirteen cohort studies involving 1,744 patients (25 independent datasets) were included. Meta-analysis revealed that HDP patients with abnormal uteroplacental and fetal Doppler indices had a significantly elevated risk of adverse perinatal outcomes (RR = 2.26, 95% CI: 1.93–2.64, *P* < 0.001). Subgroup analysis indicated that an abnormal Uterine Artery Pulsatility Index (UtA-PI) was a robust predictor (RR = 2.96, 95% CI: 2.26–3.89, *P* < 0.001, I^2^ = 6%). An abnormal Cerebroplacental Ratio (CPR) also indicated an increased risk (RR = 1.92, 95% CI: 1.58–2.33, *P* < 0.001), though with substantial heterogeneity (I^2^ = 73.0%). GRADE assessment graded the certainty of evidence as “Moderate” for the UtA-PI subgroup and “Low” for the CPR subgroup.

**Conclusion:**

Abnormal uteroplacental and fetal Doppler indices, particularly UtA-PI and CPR, are strongly associated with adverse perinatal outcomes in HDP patients. UtA-PI serves as a stable mid-trimester risk-stratification tool, while CPR functions as a valuable supplementary index in the third trimester. We recommend a multi-parametric, sequential monitoring workflow for HDP management. Future research should prioritize establishing standardized, gestational-age-specific diagnostic thresholds for CPR to enhance clinical precision.

**Systematic review registration:**

https://www.crd.york.ac.uk/PROSPERO/view/CRD420261331863, identifier CRD420261331863.

## 1 Introduction

### 1.1 Background

Hypertensive Disorders of Pregnancy (HDP) do not represent a single clinical entity but rather a continuous disease spectrum driven by progressive placental dysfunction (1). Encompassing gestational hypertension, preeclampsia, and eclampsia, HDP remains a leading cause of maternal and perinatal morbidity and mortality worldwide (2). HDP can induce defective remodeling of the spiral arteries in the placental bed and systemic vasospasm, disrupting the maternal-fetal circulatory homeostasis. This significantly increases the risk of adverse perinatal outcomes, including fetal growth restriction (FGR), preterm birth, admission to the neonatal intensive care unit (NICU), and even stillbirth (3, 4). Early identification of pregnant women on this high-risk trajectory and the implementation of timely interventions represent the core clinical imperatives to improve maternal and fetal prognoses.

Uteroplacental and fetal Doppler ultrasound, as a pivotal non-invasive tool for assessing the intrauterine environment, directly reflects placental vascular resistance and fetal hemodynamic alterations (5). Core parameters include the Uterine Artery Pulsatility Index (UtA-PI), Umbilical Artery (UA) indices, Middle Cerebral Artery (MCA) parameters, and the Cerebroplacental Ratio (CPR). UtA-PI primarily reflects the primary perfusion status of the utero-placental circulation, whereas CPR is utilized to evaluate the fetal cerebral blood flow compensatory balance under chronic hypoxia (6). Although these indices are widely applied in clinical practice, current research exhibits significant limitations: ① Discrepancies exist among individual studies; some emphasize the predictive efficacy of CPR, while others strongly advocate for the clinical value of UtA-PI or UA parameters (7, 8). ② There is a lack of rigorous, methodologically sound quantitative synthesis, rendering the relative predictive value of different parameters across the various stages of the HDP disease spectrum unclear.

### 1.2 Objective

This study aims to quantify the predictive efficacy of abnormal uteroplacental and fetal Doppler parameters for adverse perinatal outcomes in HDP patients through a systematic review and meta-analysis. Furthermore, we applied the GRADE framework to comprehensively evaluate the certainty of evidence for core indices such as UtA-PI and CPR, thereby providing a high-quality, evidence-based reference for the standardization and sequential implementation of prenatal Doppler ultrasound monitoring pathways in HDP patients.

## 2 Materials and methods

### 2.1 Literature inclusion and exclusion criteria

The inclusion criteria were strictly formulated based on the PICOS principle:

• **Population (P):** Singleton pregnant women with a confirmed diagnosis of HDP (3).

• **Exposure (E):** Evaluation of at least one uteroplacental and fetal Doppler parameter (UA, UtA, MCA, CPR, etc.) with clearly defined thresholds for abnormality.

• **Comparator (C):** HDP pregnant women with normal uteroplacental and fetal Doppler parameters.

• **Outcome (O):** Reported incidence of composite or individual adverse perinatal outcomes (typically defined in primary studies as a combination of FGR, preterm delivery, NICU admission, or perinatal death) from which Relative Risk (*RR*) and 95% Confidence Intervals (*CI*) could be extracted or calculated.

• **Study Design (S):** Non-randomized observational studies, including prospective and retrospective cohort designs.

Exclusion Criteria: ① Case reports, reviews, animal studies, and interventional randomized controlled trials (RCTs); ② Cohorts with an excessively high proportion of chronic hypertension, multiple pregnancies, or fetal structural/chromosomal anomalies; ③ Studies with ambiguous outcome definitions or missing critical data that could not be retrieved; ④ Duplicate publications.

### 2.2 Literature search strategy

This study was conducted following the PRISMA guidelines (9) and registered on the PROSPERO platform (CRD420261331863). A systematic search was performed in the Cochrane Central Register of Controlled Trials (CENTRAL), PubMed, Embase, and Science Citation Index Expanded (SCIE) databases, from January 1, 2016, to March 4, 2026, to ensure the consistency of contemporary ultrasound techniques and current HDP clinical protocols. A combination of Medical Subject Headings (MeSH) and free-text terms was utilized. Core search terms included: Hypertensive Disorders of Pregnancy, Preeclampsia, Uteroplacental and fetal Doppler, Uterine Artery Pulsatility Index, and Cerebroplacental Ratio. The reference lists of included articles were also manually screened to identify additional relevant studies.

### 2.3 Study selection and data extraction

All retrieved records were imported into EndNote 21 software for the automatic and manual removal of duplicates. Two reviewers independently conducted the initial screening of titles and abstracts, followed by full-text review. Discrepancies were resolved through consensus or consultation with a third reviewer. Data were extracted using a standardized form, capturing: first author, publication year, country, study design, sample size, baseline maternal characteristics, types of Doppler parameters and diagnostic thresholds (which were accepted as defined by the primary authors), and the frequency of adverse outcomes in each group.

### 2.4 Risk of bias and certainty of evidence assessment

The methodological quality and risk of bias of the included cohort studies were assessed independently by two reviewers using the Newcastle-Ottawa Scale (NOS) (10). Following the GRADE approach (11), the certainty of evidence for main outcomes was evaluated across five domains: risk of bias, inconsistency, indirectness, imprecision, and publication bias, and categorized as high, moderate, low, or very low.

### 2.5 Statistical analysis

Statistical analyses were performed using RevMan 5.4 (Cochrane Collaboration) and Stata 18.0 (StataCorp) software. The pooled effect size for dichotomous data was expressed as RR with its 95% CI. Statistical heterogeneity was assessed using Cochran’s Q test and the I^2^ statistic (12) (I^2^ ≥ 50% or *P* < 0.1 indicated significant heterogeneity (13), prompting the use of a random-effects model; otherwise, a fixed-effects model was employed). Subgroup analyses were conducted for parameters reported by ≥3 studies (UtA-PI, CPR). Publication bias was evaluated using funnel plots and Egger’s test (14). For indices with potential bias, a non-parametric trim-and-fill method (15) was applied for sensitivity verification. A two-sided *P* < 0.05 was considered statistically significant. It should be noted that the overall pooled analysis of all Doppler parameters was conducted strictly to illustrate the macro-level trend of association between generic hemodynamic abnormalities and adverse outcomes; precise clinical predictive values rely on the parameter-specific subgroup analyses.

## 3 Results

### 3.1 Literature screening and baseline characteristics

**FIGURE 1:**
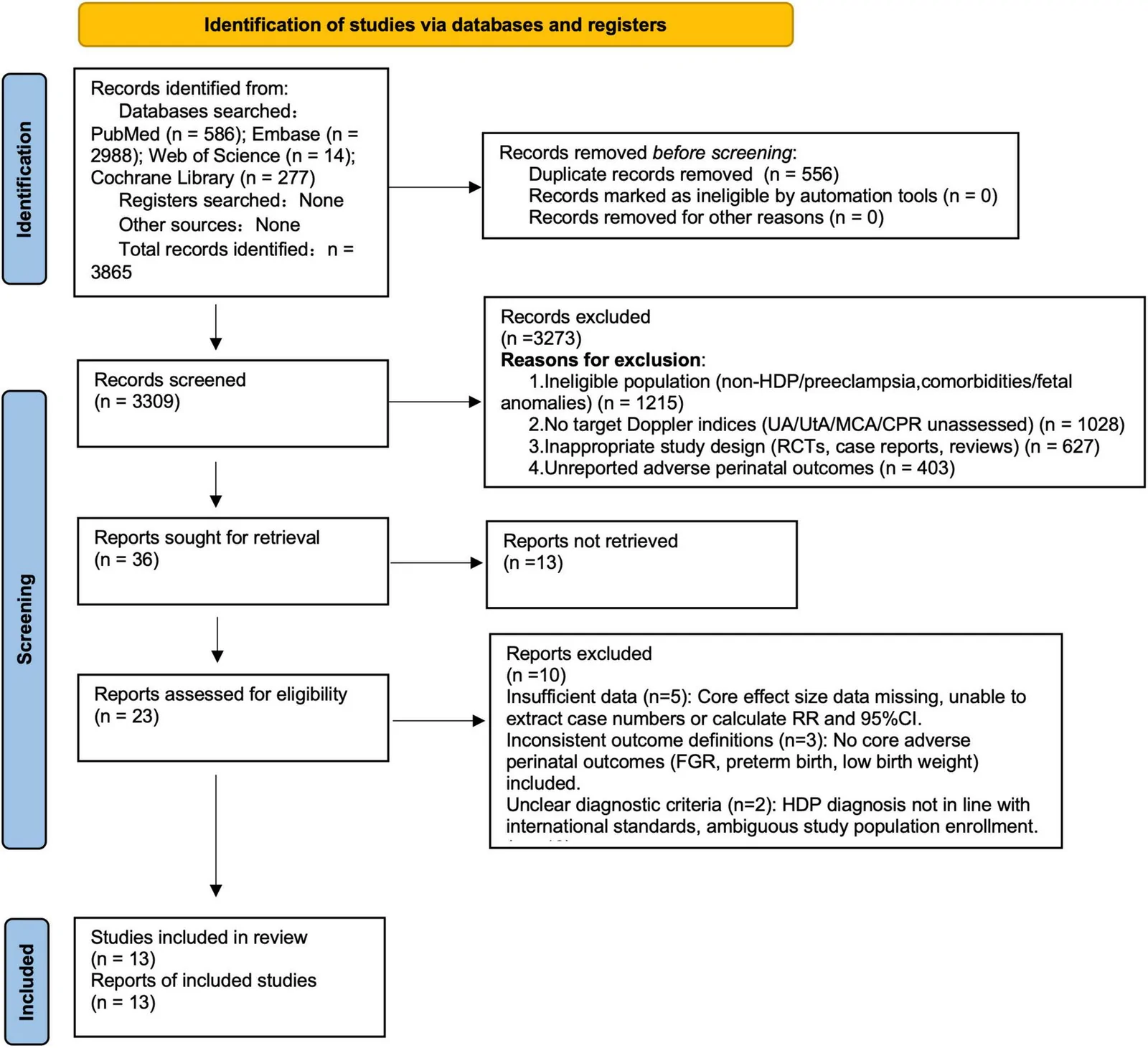
PRISMA flow diagram of the study selection process.

The initial search yielded 3,865 records (Figure 1). After a step-by-step screening process, 13 English-language cohort studies (16–28) were finally included, comprising 25 independent analyses and a cumulative sample size of 1,744 unique patients. The studies spanned multiple regions, including Asia, Europe, Africa, and Oceania. Ten were prospective cohort studies, and three were retrospective cohort studies. The majority of the included studies specified the gestational age window for ultrasound assessment (20–41 weeks) (Table 1). As detailed in Table 1, the baseline characteristics of the included cohorts encompassed the continuous spectrum of HDP. Two studies (Alanwar et al., 2018; El-Demiry et al., 2020) (16, 18)exclusively enrolled patients with severe preeclampsia (PE), while most other cohorts consisted of mixed populations (e.g., gestational hypertension, PE, and eclampsia). Several studies (such as Karabay et al., 2025; Rodríguez 2018) (19, 26) further stratified PE by onset timing (early- vs. late-onset). All included trials adopted standardized diagnostic criteria aligned with major international guidelines (e.g., ACOG (3), ISSHP, SOMANZ, and NICE). Furthermore, historical terms such as ‘mild preeclampsia’ were unified as non-severe PE in accordance with current ACOG (3) and FIGO (2) recommendations. This approach reflects routine clinical practice and provides a robust, standardized baseline for subsequent outcome evaluation.

**TABLE 1:** Characteristics of the included studies.

Author (Year)	Country	Study design	Total N	GA at US	Doppler type	Standardized HDP subclassification (n)	NOS score
Alanwar et al., (16)	Egypt	Prospective Cohort	100	34–40 weeks	CPR	Severe PE (*n* = 100)	7
Bhorat et al., (17)	South Africa	Prospective Cohort	55	NR	MPI z-score, CPR	Non-severe PE (*n* = 55)	9
El-Demiry et al., (18)	Egypt	Prospective Cohort	60	34–38 weeks	UA-PI, UA-RI, MCA-PI, MCA-RI, CPR, DV-PIVP, DV-PSV	Severe PE (*n* = 60)	9
Karabay et al., (19)	Turkey	Prospective Cohort	60	NR	AoI-IFI, AoI-S/D, AoI-PI, AoI-RI	Early-onset PE (*n* = 30); Late-onset PE (*n* = 30)	9
Loardi et al., (20)	Italy	Retrospective Cohort	311	NR	UtA-PI	Chronic HT (*n* = 97); PE (*n* = 214)	6
Lodge et al., (21)	Australia	Retrospective Cohort	258	34–37 weeks	CPR	Gestational HT (*n* = 117); PE (*n* = 141)	7
Malik et al., (22)	India	Prospective Cohort	100	32–37 weeks	CPR, UA-S/D, MCA-S/D	Gestational HT (*n* = 29); Non-severe PE (*n* = 42); Severe PE (*n* = 21); Eclampsia (*n* = 8)	6
Patil et al., (23)	India	Prospective Cohort	128	≥32 weeks	CPR	Gestational HT (*n* = 11); PE (*n* = 91); Eclampsia (*n* = 26)	7
Pérez et al., (24)	Mexico	Prospective Cohort	82	20–24 weeks	UtA-PI	Gestational HT (*n* = 64); Non-severe PE (*n* = 3); Severe PE (*n* = 15, including 2 HELLP cases)	6
Ramos et al., (25)	Brazil	Retrospective Cohort	154	26–28 weeks	UtA-PI	Late-onset PE (severity unstratified, *n* = 154)	7
Rodríguez et al., (26)	Chile	Prospective Cohort	86	≥34 weeks	UtA-PI	Late-onset non-severe PE (*n* = 33); Late-onset severe PE (*n* = 20); Late-onset PE (severity unclassified, *n* = 33)	9
Saxena and Alka, (27)	India	Prospective Cohort	150	28–41 weeks	CPR	Gestational HT (*n* = 88); PE (*n* = 62)	7
Shireesha, (28)	India	Prospective Cohort	200	≥30 weeks	CPR	Gestational HT and PE (subgroup sizes unreported, *n* = 200)	7

GA at US, gestational age at ultrasound examination; CPR, cerebroplacental ratio; MPI, myocardial performance index; UA-PI, umbilical artery pulsatility index; UA-RI, umbilical artery resistance index; UA-S/D, umbilical artery systolic/diastolic ratio; MCA-PI, middle cerebral artery pulsatility index; MCA-RI, middle cerebral artery resistance index; MCA-S/D, middle cerebral artery systolic/diastolic ratio; DV-PIVP, ductus venosus pulsatility index for veins; DV-PSV, ductus venosus peak systolic velocity; AoI-IFI, aortic isthmus intrafetal flow index; AoI-S/D, aortic isthmus systolic/diastolic ratio; AoI-PI, aortic isthmus pulsatility index; AoI-RI, aortic isthmus resistance index; NR, not reported. HDP, hypertensive disorders of pregnancy; HT, hypertension; PE, preeclampsia; IUGR, intrauterine growth restriction.

### 3.2 Methodological quality assessment

The NOS evaluation revealed that 10 studies had a low risk of bias (score ≥ 7), and 3 had a moderate risk of bias (score = 6) (Table 2). Point deductions in moderate-risk studies were primarily due to inadequate control of confounding factors between groups. Overall, the methodological quality of the included literature was sound, with no high-risk studies identified.

**TABLE 2:** Quality Assessment of Included Studies (NOS).

Study	Design	Selection (4*)	Comparability (2*)	Outcome/Exposure (3*)	Total Score	Quality
Alanwar et al., (16)	Cohort	4	1	2	7	High
Bhorat et al., (17)	Cohort	4	2	3	9	High
El-Demiry et al., (18)	Cohort	4	2	3	9	High
Lodge et al., (21)	Cohort	3	2	2	7	High
Loardi et al., (20)	Cohort	3	1	2	6	Moderate
Malik et al., (22)	Cohort	4	1	1	6	Moderate
Patil et al., (23)	Cohort	4	1	2	7	High
Pérez et al., (24)	Cohort	3	1	2	6	Moderate
Ramos et al. (25)	Cohort	3	2	2	7	High
Rodríguez et al., (26)	Cohort	4	2	3	9	High
Saxena and Alka, (27)	Cohort	4	1	2	7	High
Shireesha, (28)	Cohort	4	1	2	7	High
Karabay et al., (19)	Cohort	4	2	3	9	High

The Newcastle-Ottawa Scale (NOS) was used to assess the methodological quality of cohort studies. The maximum score is 9, and a score ≥7 indicates high-quality studies.

### 3.3 Meta-analysis results

**FIGURE 2:**
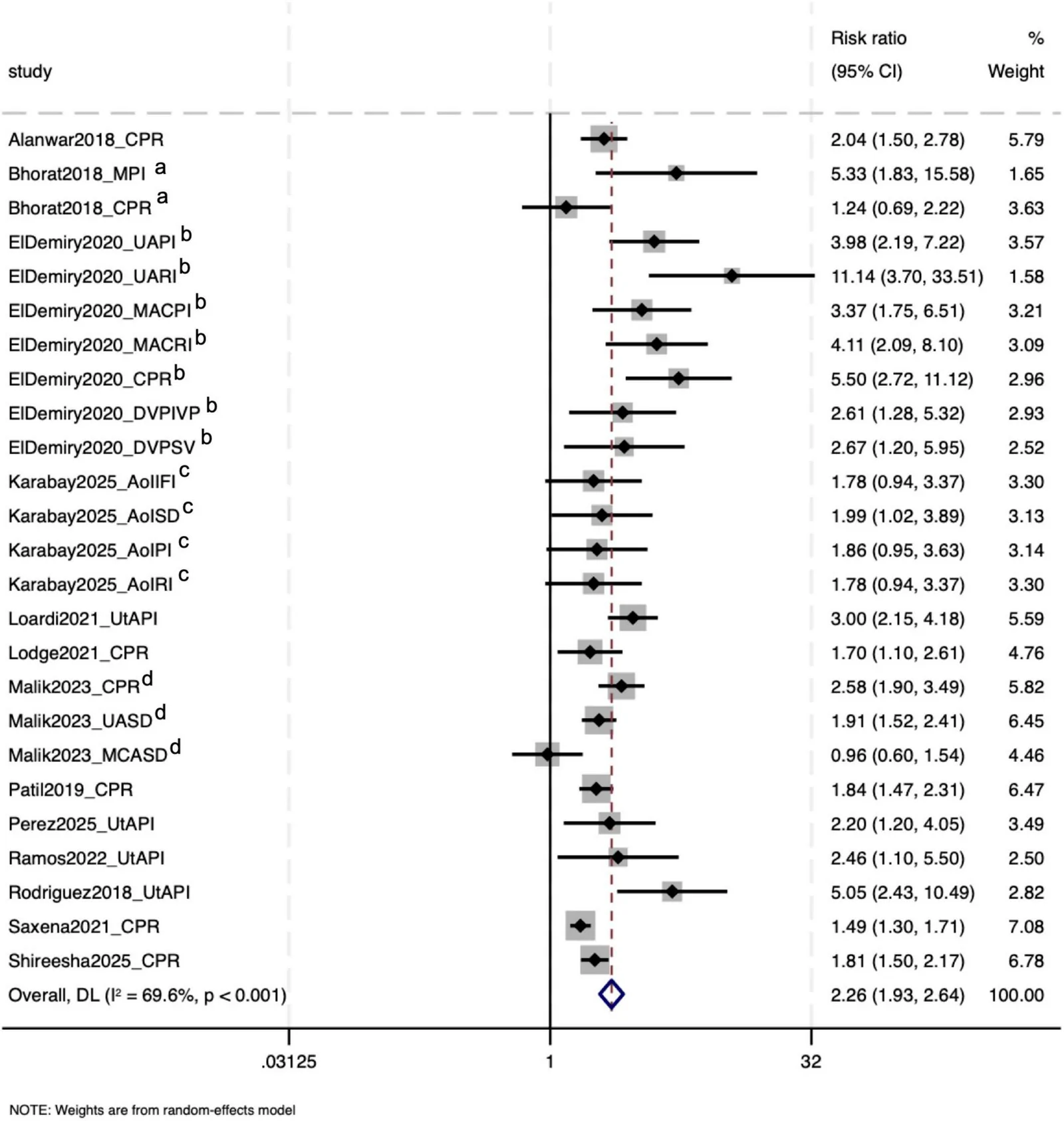
Forest plot of the overall meta-analysis assessing the risk of adverse perinatal outcomes in HDP patients with normal versus abnormal uteroplacental and fetal Doppler indices.

*Overall Parameter Analysis:* Pooled analysis based on a random-effects model demonstrated that HDP pregnant women with abnormal uteroplacental and fetal Doppler indices had a significantly higher risk of experiencing adverse perinatal outcomes compared to those with normal parameters (*RR* = 2.26, 95% *CI*: 1.93–2.64, *P* < 0.001; Figure 2). Substantial heterogeneity was observed in the overall analysis (*I*^2^ = 69.6%, *P* < 0.001). However, the overall pooled *RR* should be interpreted with caution due to potential unit-of-analysis overlaps. As annotated by superscripts (a–d) in Figure 2, multiple Doppler parameters derived from the same patient cohorts were included, which could overestimate the sample size and effect sizes. Therefore, our primary clinical conclusions are strictly drawn from the parameter-specific subgroup analyses.

**FIGURE 3:**
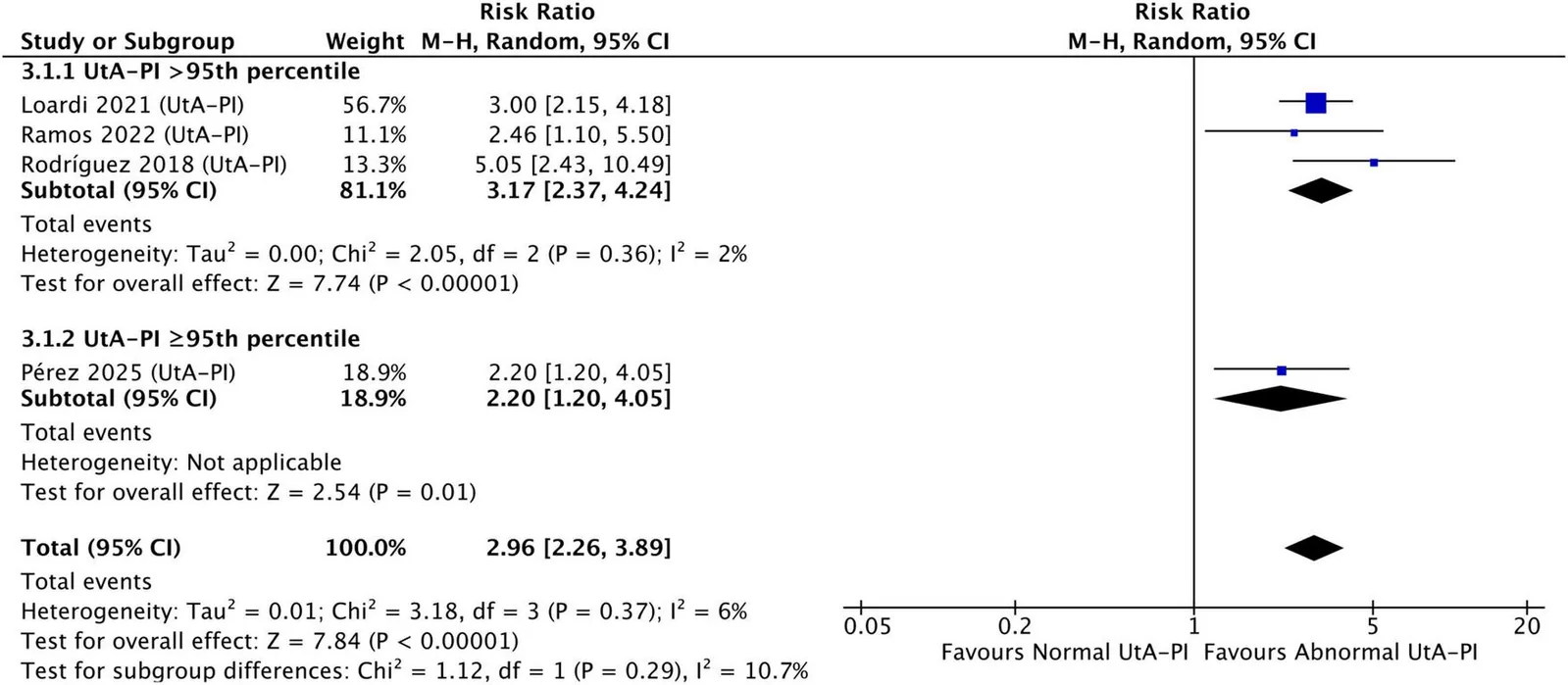
Forest plot of the subgroup meta-analysis evaluating the predictive value of abnormal Uterine Artery Pulsatility Index (UtA-PI).

**FIGURE 4:**
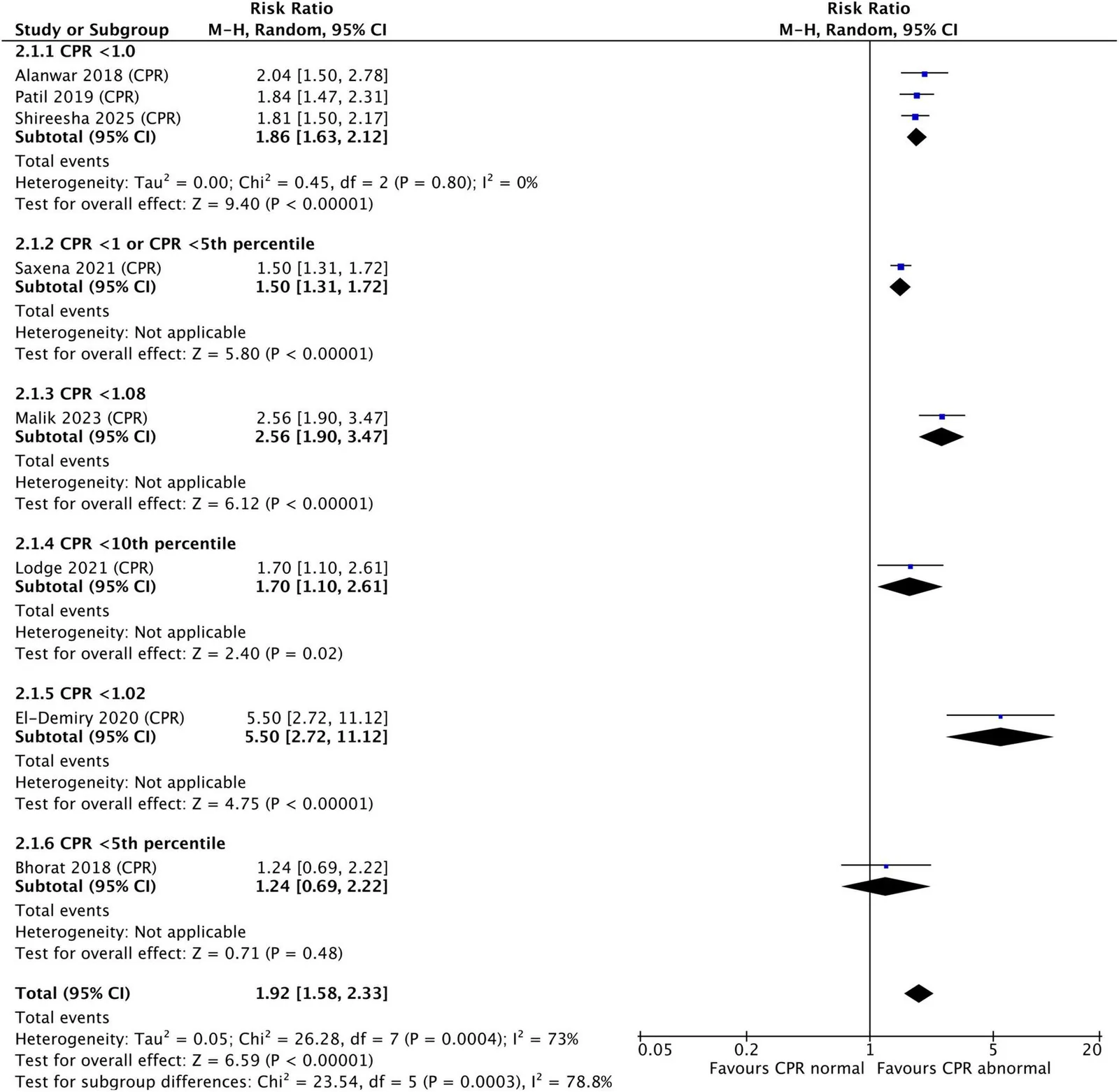
Forest plot of the subgroup meta-analysis evaluating the predictive value of abnormal Cerebroplacental Ratio (CPR).

*Core Parameter Subgroup Analysis:* Within the evaluated subgroups, abnormal UtA-PI demonstrated a highly significant predictive efficacy for adverse outcomes (*RR* = 2.96, 95% *CI*: 2.26–3.89, *P* < 0.001; Figure 3). Notably, this subgroup exhibited remarkably low intra-group heterogeneity (*I*^2^ = 6%, *P* = 0.37), suggesting high statistical consistency. However, as with all observational data, this low statistical heterogeneity does not preclude the potential presence of underlying methodological biases or clinical confounding. Similarly, an abnormal CPR significantly increased the risk of adverse perinatal outcomes (*RR* = 1.92, 95% *CI*: 1.58–2.33, *P* < 0.001; Figure 4). However, the CPR subgroup exhibited substantial statistical heterogeneity (*I*^2^ = 73.0%, *P* = 0.0004), suggesting the presence of complex clinical confounders.

(Note: One included study (Karabay et al., 2025) (19) evaluated isolated fetal aortic isthmus Doppler indices; this study was incorporated into the overall analysis but excluded from the parameter-specific subgroup analyses as it did not meet the predefined threshold of ≥ 3 studies per index.)

### 3.4 Publication bias and sensitivity analysis

**FIGURE 5:**
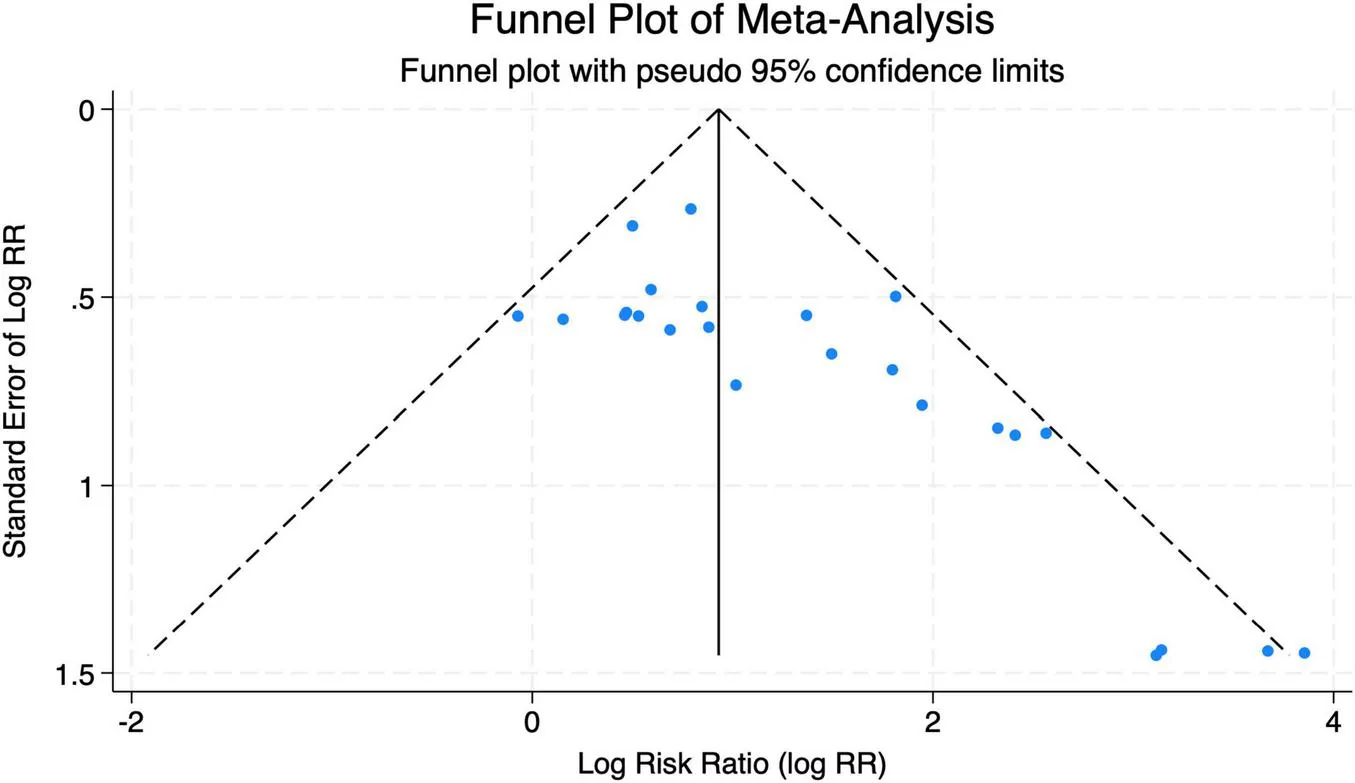
Funnel plot for assessing potential publication bias.

Visual inspection of the funnel plot for the overall analysis suggested mild asymmetry (Figure 5), and Egger’s linear regression test indicated the presence of small-study effects (*P* < 0.001). To visually demonstrate this issue as recommended, we conducted a stratified analysis based on sample size (Supplementary Figure 1). Single-center small-sample studies (*N* < 100) exhibited a notably higher pooled effect size (RR = 2.87, 95% CI: 2.19–3.75) compared to large-scale cohorts (*N* ≥ 100, RR = 1.88, 95% CI: 1.61–2.21). This confirms that smaller cohorts tended to overestimate the predictive effects, thereby driving the observed funnel plot asymmetry. Subsequent sensitivity analysis using the leave-one-out method demonstrated that the exclusion of any single study did not substantively alter the pooled effect sizes. Furthermore, the non-parametric trim-and-fill analysis revealed that the number of missing studies required to be imputed was zero, and the adjusted pooled RR (2.26, 95% CI: 1.93–2.64) remained identical to the original calculation. This confirms that the core effect sizes of this study were not severely skewed by the potential non-publication of negative results, reflecting a high degree of reliability.

### 3.5 GRADE certainty of evidence

According to the GRADE criteria (11), the certainty of evidence for both the overall analysis and the UtA-PI subgroup initially defaulted to ‘Low‘ given the observational nature of the included cohorts. We subsequently upgraded this rating to ‘Moderate’ due to several methodological strengths. Specifically, the pooled effect magnitude was notably large (*RR* > 2.0), reflecting a strong clinical association between abnormal Doppler parameters and adverse perinatal outcomes. Furthermore, the leave-one-out analysis confirmed that no single study skewed the findings, suggesting limited residual confounding from variables such as HDP severity or baseline gestational age. The consistency of this predictive trend across diverse populations, combined with the absence of publication bias in Egger’s and trim-and-fill tests (14, 15), fully justified the upward adjustment. Conversely, the evidence certainty for the CPR subgroup remained ‘Low’. Its pooled effect size did not meet the upgrade threshold, and substantial inter-study heterogeneity persisted, precluding any further adjustments (Table 3).

**TABLE 3:** GRADE summary of findings for adverse perinatal outcomes.

Uteroplacental and fetal Doppler parameter	No. of studies	Risk of bias	Inconsistency	Indirectness	Imprecision	Publication bias	Relative effect (95% CI)	Certainty of evidence
Overall abnormal parameters	13	Not serious	Serious	Not serious	Not serious	Not serious	RR 2.26 (1.93–2.64)	Moderate
Abnormal UtA-PI	4	Not serious	Not serious	Not serious	Not serious	Not serious	RR 2.96 (2.26–3.89)	Moderate
Abnormal CPR	8	Not serious	Serious	Not serious	Not serious	Not serious	RR 1.92 (1.58–2.33)	Low

GRADE Working Group grades of evidence: Moderate certainty: We are moderately confident in the effect estimate. Low certainty: Our confidence in the effect estimate is limited. a. Substantial statistical heterogeneity was observed (I^2^ > 50%). b. Started at ‘Low‘ due to observational study design, but upgraded one level to ‘Moderate‘ due to a large magnitude of effect (RR > 2.0). c. Started at ‘Low‘ due to observational study design, but upgraded one level to ‘Moderate’ due to a large magnitude of effect (RR > 2.0) and no serious inconsistency (I^2^ = 6%). d. Started at ‘Low’ due to observational study design. No upgrade was performed as the pooled effect size did not meet the robust threshold for upgrade, and substantial inter-study heterogeneity (I^2^ = 73.0%) persisted.

## 4 Discussion

### 4.1 Clinical value of core findings

This systematic review, synthesizing 13 high-quality cohort studies, demonstrates that abnormal uteroplacental and fetal Doppler parameters are strongly associated with adverse perinatal outcomes and serve as valuable risk stratification biomarkers in HDP pregnant women. Among the evaluated indices, UtA-PI exhibited exceptional predictive stability (RR = 2.96) with minimal heterogeneity, highlighting its value in identifying primary placental perfusion injury. Abnormal CPR was also correlated with adverse outcomes (RR = 1.92), but presented high heterogeneity, constrained by varying clinical cutoffs and study population differences. The GRADE evidence stratification further provides an objective benchmark for the confidence in applying these indices clinically.

### 4.2 Pathophysiological and clinical interpretations

The evolution of Doppler parameters essentially mirrors the classic two-stage pathophysiological cascade of HDP. Primarily, defective remodeling of the utero-placental spiral arteries leads to placental ischemia, which manifests clinically as elevated UtA-PI resistance. Subsequently, this primary placental insufficiency triggers systemic endothelial dysfunction and the overt clinical syndrome of HDP (29). Notably, this anatomical perfusion deficit is frequently accompanied by the excessive release of anti-angiogenic factors (e.g., sFlt-1) and endothelial cell injury (30). The abnormality of Doppler indices (functional level) shares a homologous origin with the imbalance of angiogenic factors (biochemical level), providing a solid pathological foundation for the large effect size observed for UtA-PI in this population. When placental functional impairment progresses to a critical threshold, the fetus triggers the “brain-sparing effect,” prioritizing blood supply to the central nervous system by reducing MCA resistance, which manifests as a decreased CPR (6). By integrating the waveforms of the UA and DV, clinicians can construct a comprehensive, longitudinal “maternal-placental-fetal” hemodynamic evaluation model, clearly mapping the logical trajectory from proximal ischemia at the origin to fetal multi-organ decompensation.

Additionally, the emerging clinical utility of angiogenic biomarkers, such as the sFlt-1/PlGF ratio, warrants consideration alongside Doppler parameters (30). Uteroplacental and fetal Doppler indices (functional/hemodynamic indicators) and angiogenic biomarkers (molecular indicators of endothelial dysfunction) are not mutually exclusive but highly complementary. Angiogenic imbalance leads to maldevelopment of the placental vasculature, which manifests upstream as an elevated UtA-PI. Future research should evaluate “ultrasound-biochemical joint models,” utilizing biomarkers to identify high-risk individuals and Doppler indices like CPR to dynamically monitor subsequent fetal decompensation, thereby constructing a holistic predictive framework from molecular alteration to functional compromise.

### 4.3 Temporal dynamics of assessment timing

Although the demographic reporting formats of the primary literature precluded precise quantitative subgroup analyses based on strict gestational age, a review of the literature revealed significant temporal distribution patterns in clinical application windows. Studies evaluating UtA-PI were predominantly concentrated in the second to early third trimesters, whereas CPR investigations were mainly focused on the late third trimester (≥32 or ≥34 weeks). This temporal distribution perfectly aligns with the clinical disease trajectory of HDP: mid-trimester UtA-PI abnormality reflects early placental vascular damage, whereas third-trimester CPR decline indicates fetal hemodynamic compensation to chronic hypoxia. These two indices are fundamentally complementary, creating a complete pathophysiological and chronological closed loop for disease progression monitoring (2).

### 4.4 Deconstructing heterogeneity and publication bias

To quantitatively explore the sources of the substantial heterogeneity in the CPR subgroup (I^2^ = 73.0%), we conducted stratified subgroup analyses (Supplementary Figures 2 and 3). Stratification by disease severity revealed that cohorts exclusively enrolling pure preeclampsia patients exhibited a higher pooled effect size (RR = 2.32, 95% CI: 1.17–4.62) compared to mixed HDP populations (RR = 1.82, 95% CI: 1.51–2.18). Subsequent stratification by ultrasound gestational age demonstrated that studies with strict late third-trimester assessments (≥32 weeks) yielded a higher pooled RR of 2.23 (95% CI: 1.72–2.90) with intra-group heterogeneity reduced to I^2^ = 64%, whereas studies with variable timing reported a lower RR of 1.59 (I^2^ = 41%). These quantitative findings indicate that clinical phenotypic variation and unstandardized assessment timing contribute to the statistical variance. Furthermore, as discussed below, a primary driver of this heterogeneity is the lack of standardized diagnostic thresholds. Currently, there is no universally accepted, gestational-age-specific CPR cutoff specifically tailored for HDP populations. The concurrent use of fixed empirical thresholds (e.g., <1.0) and varying percentile cutoffs across different cohorts inevitably introduces diagnostic misclassification, artificially inflating statistical variance (31). Furthermore, inherent clinical population differences play a critical role. The included cohorts encompassed a broad spectrum of disease severity, ranging from mild gestational hypertension to severe preeclampsia. This phenotypic variation, compounded by differing gestational age windows at the time of Doppler assessment and diverse ethnic or nutritional backgrounds, inherently alters the baseline fetoplacental hemodynamics and, consequently, the predictive sensitivity of CPR. Methodological discrepancies also contribute to the overall variance, particularly regarding differences in ultrasound equipment calibration and inter-operator measurement variability. Additionally, aggressive iatrogenic interventions triggered by severely abnormal Doppler findings may artificially decouple the predictor from natural outcomes, a recognized challenge in observational obstetrics research (32). The significant Egger’s test (*P* < 0.001) combined with a zero-imputation trim-and-fill result suggests that the funnel plot asymmetry is likely driven by ‘small-study effects’ – where smaller, specialized cohorts reported exaggerated positive effect sizes due to more severe HDP phenotypes or aggressive clinical protocols—rather than true publication bias.

### 4.5 Strengths, limitations, and long-term prognosis

The strengths of this study lie in its strict adherence to PRISMA guidelines, the use of the non-parametric trim-and-fill method to eliminate bias interference, and the application of the GRADE framework to provide evidence-based ratings for ultrasound indices in this field. Limitations include: the inherent inability of observational designs to establish absolute causality, meaning that unmeasured confounding factors, selection biases, and potential publication biases may still influence the pooled estimates; and the insufficient granularity of data reported in primary literature, which restricted meta-regression analyses based on specific gestational ages or early/late-onset preeclampsia subtypes.

Furthermore, while this study focused primarily on immediate perinatal outcomes, the long-term neurodevelopmental impact of fetal hemodynamic alterations warrants consideration. The ‘brain-sparing effect,’ characterized by a reduced CPR, serves as an evolutionary compensatory mechanism to prioritize cerebral blood supply under chronic hypoxia (6). However, clinical evidence indicates this redistribution is a double-edged sword. Prolonged cerebral hyperperfusion paired with chronic low-grade hypoxia can disrupt axonal guidance and induce subtle microstructural white matter injury in the developing brain. Clinically, follow-up data from the TRUFFLE trial demonstrate that short-term survival or hemodynamic optimization does not inherently guarantee long-term neurological preservation (33). Children with a reduced prenatal CPR exhibit higher risks of cognitive deficits and motor delays at school age. Thus, a low CPR should be interpreted as a critical early warning of prolonged intrauterine stress rather than an absolute neuroprotective shield, underscoring the need for future Doppler research to extend follow-up horizons into childhood. Additionally, as demonstrated by our stratification, single-center small-sample studies exhibited an overestimation bias in predicting adverse outcomes. Consequently, the clinical application of these predictive indices from smaller cohorts should be interpreted with caution, and future guidelines must prioritize large-scale, multicenter validations.

### 4.6 Clinical implications and future directions

Integrating our meta-analytic results and GRADE evidence synthesis, we propose a specific, multi-parametric, longitudinal, and sequential uteroplacental and fetal Doppler monitoring pathway for the clinical management of pregnancies complicated by HDP:

∙ 20–24 weeks (Mid-trimester): Implement UtA-PI screening as the preferred first-line indicator for early risk stratification of placental vascular damage.

∙≥32 weeks (Late third trimester): Utilize dynamic CPR monitoring as a supplementary index to evaluate impending fetal cerebral compensatory reserve under chronic hypoxia.

**FIGURE 6:**
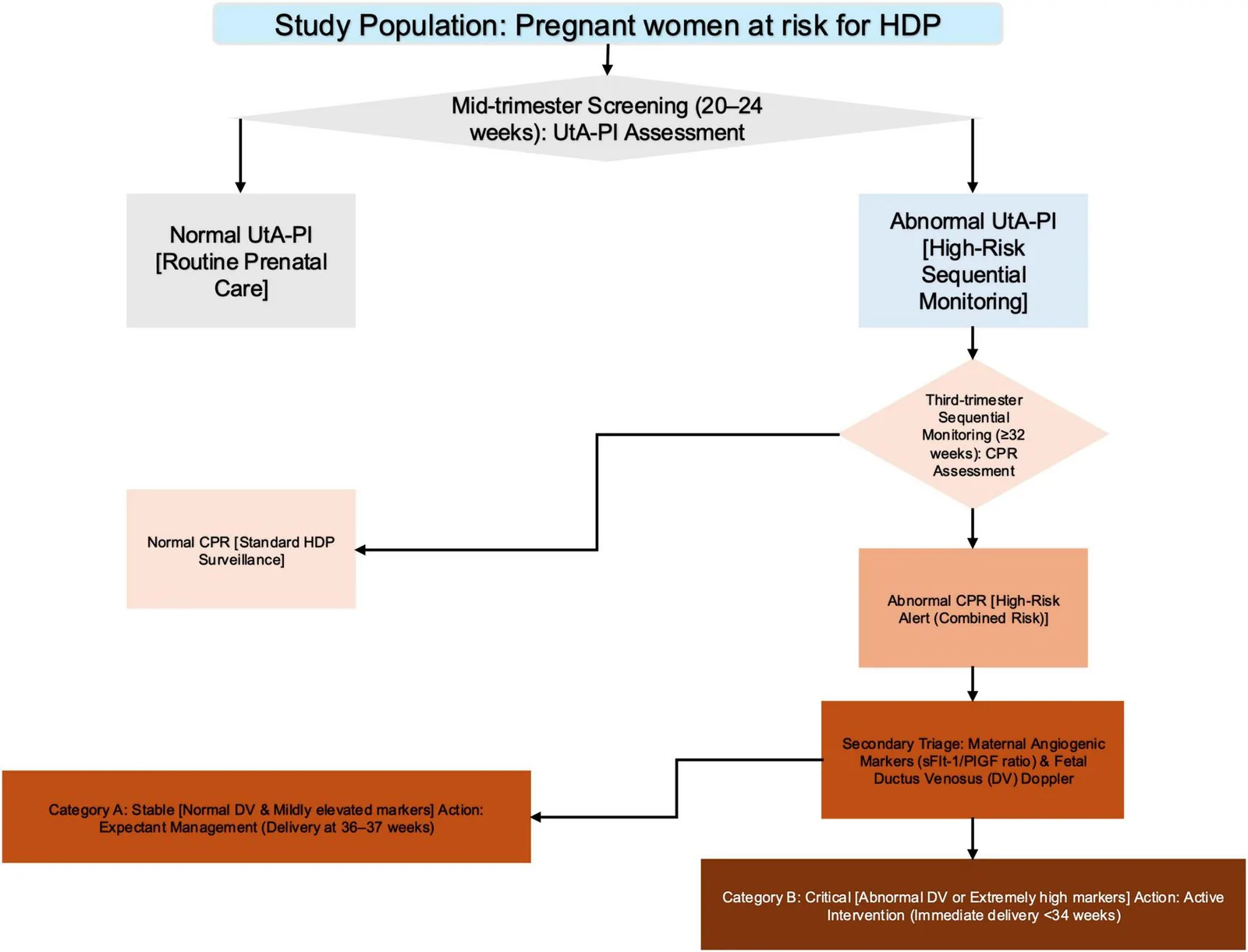
Proposed multi-parametric, sequential uteroplacental and fetal Doppler monitoring workflow for the clinical management of pregnancies complicated by HDP.

∙ Concurrent Abnormalities: If both elevated UtA-PI and decreased CPR occur simultaneously, intensified antenatal surveillance is mandated. While traditional guidelines [e.g., ACOG (3), ISUOG (5)] often rely on reactive symptom monitoring, our study outlines a proactive, graded intervention algorithm integrating secondary tools, including the sFlt-1/PlGF ratio and ductus venosus (DV) Doppler, to optimally balance the competing risks of intrauterine fetal demise and iatrogenic preterm birth (34) (Figure 6). This multi-parametric approach allows for stratified delivery timing – ranging from expectant management (36–37 weeks) for stable cases to active intervention (<34 weeks post-corticosteroids) for severe placental insufficiency – thereby maximizing perinatal outcomes while minimizing iatrogenic prematurity.

Because current CPR evidence quality remains “Low” due to inconsistent cutoffs, future research must prioritize large-scale, multicenter prospective cohorts to establish HDP-specific, gestational-age-adjusted CPR reference curves (e.g., utilizing Z-scores or Multiples of the Median [MoM]). Such standardization is crucial to enhance cross-study comparability and provide a more precise basis for individualized interventions in high-risk pregnancies.

## 5 Conclusion

Abnormal uteroplacental and fetal Doppler indices are strongly associated with adverse perinatal outcomes and serve as valuable risk stratification biomarkers in HDP pregnant women. Among these, the Uterine Artery Pulsatility Index (UtA-PI) demonstrates extremely high stability and “Moderate” quality evidence, acting as a key monitoring tool for early clinical risk stratification. The Cerebroplacental Ratio (CPR) also holds significant clinical value; however, constrained by unstandardized diagnostic cutoffs, its current evidence quality is “Low”. Optimizing HDP management urgently requires establishing standardized, gestational-age-adjusted CPR reference criteria through multicenter prospective cohorts. Implementing a multi-parametric, sequential monitoring workflow will provide a precise framework for determining appropriately timed delivery in high-risk pregnancies.
