# Supplementary material for: The predictive value of abnormal uteroplacental and fetal Doppler indices for adverse perinatal outcomes in hypertensive disorders of pregnancy: a systematic review and meta-analysis
Source: Front Med (Lausanne). 2026 Aug 12;13:1923180. doi: 10.3389/fmed.2026.1923180 (PMC13506527; doi:10.3389/fmed.2026.1923180)
Supplement: Supplementary file 1 [file Data_Sheet_1.pdf]

PRISMA 2020 Checklist

Section and Topic	Item #	Checklist item	Location where item is reported
**TITLE**			
Title	1	Identify the report as a systematic review.	Title page
**ABSTRACT**			
Abstract	2	See the PRISMA 2020 for Abstracts checklist.	Abstract
**INTRODUCTION**			
Rationale	3	Describe the rationale for the review in the context of existing knowledge.	Section 1.1. Background
Objectives	4	Provide an explicit statement of the objective(s) or question(s) the review addresses.	Section 1.2. Objective
**METHODS**			
Eligibility criteria	5	Specify the inclusion and exclusion criteria for the review and how studies were grouped for the syntheses.	Section 2.1. Literature Inclusion and Exclusion Criteria
Information sources	6	Specify all databases, registers, websites, organisations, reference lists and other sources searched or consulted to identify studies. Specify the date when each source was last searched or consulted.	Section 2.2. Literature Search Strategy
Search strategy	7	Present the full search strategies for all databases, registers and websites, including any filters and limits used.	Section 2.2. Literature Search Strategy
Selection process	8	Specify the methods used to decide whether a study met the inclusion criteria of the review, including how many reviewers screened each record and each report retrieved, whether they worked independently, and if applicable, details of automation tools used in the process.	Section 2.3. Study Selection and Data Extraction
Data collection process	9	Specify the methods used to collect data from reports, including how many reviewers collected data from each report, whether they worked independently, any processes for obtaining or confirming data from study investigators, and if applicable, details of automation tools used in the process.	PSection 2.3. Study Selection and Data Extraction
Data items	10a	List and define all outcomes for which data were sought. Specify whether all results that were compatible with each outcome domain in each study were sought (e.g. for all measures, time points, analyses), and if not, the methods used to decide which results to collect.	Section 2.1. Literature Inclusion and Exclusion Criteria (Outcome)
	10b	List and define all other variables for which data were sought (e.g. participant and intervention characteristics, funding sources). Describe any assumptions made about any missing or unclear information.	Section 2.3. Study Selection and Data Extraction
Study risk of bias assessment	11	Specify the methods used to assess risk of bias in the included studies, including details of the tool(s) used, how many reviewers assessed each study and whether they worked independently, and if applicable, details of automation tools used in the process.	Section 2.4. Risk of Bias and Certainty of Evidence Assessment
Effect measures	12	Specify for each outcome the effect measure(s) (e.g. risk ratio, mean difference) used in the synthesis or presentation of results.	Section 2.5. Statistical Analysis
Synthesis methods	13a	Describe the processes used to decide which studies were eligible for each synthesis (e.g. tabulating the study intervention characteristics and comparing against the planned groups for each synthesis (item #5)).	Section 2.5. Statistical Analysis

PRISMA 2020 Checklist

Section and Topic	Item #	Checklist item	Location where item is reported
	13b	Describe any methods required to prepare the data for presentation or synthesis, such as handling of missing summary statistics, or data conversions.	Section 2.5. Statistical Analysis
	13c	Describe any methods used to tabulate or visually display results of individual studies and syntheses.	Section 2.5. Statistical Analysis
	13d	Describe any methods used to synthesize results and provide a rationale for the choice(s). If meta-analysis was performed, describe the model(s), method(s) to identify the presence and extent of statistical heterogeneity, and software package(s) used.	Section 2.5. Statistical Analysis
	13e	Describe any methods used to explore possible causes of heterogeneity among study results (e.g. subgroup analysis, meta-regression).	Section 2.5. Statistical Analysis
	13f	Describe any sensitivity analyses conducted to assess robustness of the synthesized results.	Section 2.5. Statistical Analysis
Reporting bias assessment	14	Describe any methods used to assess risk of bias due to missing results in a synthesis (arising from reporting biases).	Section 2.5. Statistical Analysis
Certainty assessment	15	Describe any methods used to assess certainty (or confidence) in the body of evidence for an outcome.	Section 2.4. Risk of Bias and Certainty of Evidence Assessment
**RESULTS**			
Study selection	16a	Describe the results of the search and selection process, from the number of records identified in the search to the number of studies included in the review, ideally using a flow diagram.	Section 3.1. Literature Screening and Baseline Characteristics; Figure 1
	16b	Cite studies that might appear to meet the inclusion criteria, but which were excluded, and explain why they were excluded.	Section 3.1. Literature Screening and Baseline Characteristics
Study characteristics	17	Cite each included study and present its characteristics.	Table 1: Characteristics of the Included Studies
Risk of bias in studies	18	Present assessments of risk of bias for each included study.	Section 3.2. Methodological Quality Assessment; Table 2
Results of individual studies	19	For all outcomes, present, for each study: (a) summary statistics for each group (where appropriate) and (b) an effect estimate and its precision (e.g. confidence/credible interval), ideally using structured tables or plots.	Section 3.3. Meta-Analysis Results
Results of syntheses	20a	For each synthesis, briefly summarise the characteristics and risk of bias among contributing studies.	Section 3.3. Meta-Analysis Results
	20b	Present results of all statistical syntheses conducted. If meta-analysis was done, present for each the summary estimate and its precision (e.g. confidence/credible interval) and measures of statistical heterogeneity. If comparing groups, describe the direction of the	Section 3.3. Meta-Analysis

PRISMA 2020 Checklist

Section and Topic	Item #	Checklist item	Location where item is reported
		effect.	Results
	20c	Present results of all investigations of possible causes of heterogeneity among study results.	Section 3.3. Meta-Analysis Results
	20d	Present results of all sensitivity analyses conducted to assess the robustness of the synthesized results.	Section 3.3. Meta-Analysis Results
Reporting biases	21	Present assessments of risk of bias due to missing results (arising from reporting biases) for each synthesis assessed.	Section 3.4. Publication Bias and Sensitivity Analysis; Figure 5
Certainty of evidence	22	Present assessments of certainty (or confidence) in the body of evidence for each outcome assessed.	Section 3.5. GRADE Certainty of Evidence; Table 3
**DISCUSSION**			
Discussion	23a	Provide a general interpretation of the results in the context of other evidence.	Section 4.1. Clinical Value of Core Findings
	23b	Discuss any limitations of the evidence included in the review.	Section 4.5. Strengths, Limitations, and Long-term Prognosis
	23c	Discuss any limitations of the review processes used.	Section 4.5. Strengths, Limitations, and Long-term Prognosis
	23d	Discuss implications of the results for practice, policy, and future research.	Section 4.6. Clinical Implications and Future Directions
**OTHER INFORMATION**			
Registration and protocol	24a	Provide registration information for the review, including register name and registration number, or state that the review was not registered.	Abstract; Section 2.2. Literature Search Strategy (PROSPERO CRD420261331863)
	24b	Indicate where the review protocol can be accessed, or state that a protocol was not prepared.	Section 2.2. Literature Search Strategy
	24c	Describe and explain any amendments to information provided at registration or in the protocol.	Not applicable
Support	25	Describe sources of financial or non-financial support for the review, and the role of the funders or sponsors in the review.	Declarations

Section and Topic	Item #	Checklist item	Location where item is reported
			(Funding)
Competing interests	26	Declare any competing interests of review authors.	Declarations (Competing interests)
Availability of data, code and other materials	27	Report which of the following are publicly available and where they can be found: template data collection forms; data extracted from included studies; data used for all analyses; analytic code; any other materials used in the review.	Declarations (Availability of data and materials)

From: Page MJ, McKenzie JE, Bossuyt PM, Boutron I, Hoffmann TC, Mulrow CD, et al. The PRISMA 2020 statement: an updated guideline for reporting systematic reviews. BMJ 2021;372:n71. doi: 10.1136/bmj.n71. This work is licensed under CC BY 4.0. To view a copy of this license, visit https://creativecommons.org/licenses/by/4.0/

PROSPERO
International prospective register of systematic reviews

The Predictive Value of Fetal Doppler Parameters for Adverse Perinatal Outcomes in Hypertensive Disorders of Pregnancy: A Systematic Review and Meta-Analysis

Peng Zhou, Siyuan Li

Citation
Peng Zhou, Siyuan Li. The Predictive Value of Fetal Doppler Parameters for Adverse Perinatal Outcomes in Hypertensive Disorders of Pregnancy: A Systematic Review and Meta-Analysis. PROSPERO 2026 CRD420261331863. Available from https://www.crd.york.ac.uk/PROSPERO/view/CRD420261331863.

REVIEW TITLE AND BASIC DETAILS

Review title

The Predictive Value of Fetal Doppler Parameters for Adverse Perinatal Outcomes in Hypertensive Disorders of Pregnancy: A Systematic Review and Meta-Analysis

Condition or domain being studied 1 change

Pre-eclampsia; Hypertension; Obstetric Uterine Artery Doppler; Ultrasound Doppler Abnormal; Fetal Heart Monitoring; High Risk Pregnancy; Risk Assessment; Birth Outcome; Perinatal morbidity; Pregnancy complication; Prognosis

This review aims to assess the predictive value of abnormal fetal Doppler ultrasound parameters (including uterine artery, umbilical artery, middle cerebral artery indices, and cerebroplacental ratio) for adverse perinatal outcomes in pregnant women with hypertensive disorders of pregnancy, including pre-eclampsia and gestational hypertension.

Rationale for the review 1 change

Hypertensive disorders in pregnancy (HDP), such as pre-eclampsia, are leading causes of adverse pregnancy outcomes for mothers and infants globally. In clinical practice, fetal Doppler ultrasound (including umbilical artery, middle cerebral artery, ductus venosus, etc.) is a routine tool to assess fetal hemodynamics and uteroplacental function, and abnormal fetal Doppler blood flow indices are considered potential predictors of adverse perinatal outcomes in HDP patients. However, individual studies have reported inconsistent findings, and there is a lack of an updated, comprehensive systematic

synthesis to confirm the clinical value of these indicators. This systematic review and meta-analysis will pool existing evidence on the association between abnormal fetal Doppler blood flow and adverse pregnancy outcomes in HDP pregnancies. It will clarify the prognostic role of fetal Doppler assessment in this population, provide evidence-based support for clinical risk stratification and management, and fill the gap in current knowledge about this monitoring method in hypertensive pregnancies.

Review objectives 1 change

To systematically evaluate and quantify the predictive value of fetal Doppler parameters (including umbilical artery, uterine artery, middle cerebral artery indices, and cerebroplacental ratio) for adverse perinatal outcomes in women with hypertensive disorders of pregnancy.

Keywords 1 change

Preeclampsia; Hypertensive disorders of pregnancy; Fetal Doppler ultrasound; Umbilical artery Doppler; Uterine artery Doppler; Adverse perinatal outcomes

Country

China

ELIGIBILITY CRITERIA

Population 1 change

Included

1. Study design: prospective or retrospective observational cohort studies.

2. Participants: singleton pregnant women diagnosed with hypertensive disorders of pregnancy (HDP).

3. Exposure: at least one fetal Doppler index measured, including umbilical artery (UA), uterine artery (UtA), middle cerebral artery (MCA), cerebroplacental ratio (CPR), with clear abnormal cutoff values.

4. Outcomes: reported composite or individual adverse perinatal outcomes, with sufficient data to extract relative risk (RR) and 95% confidence interval (CI).

5. Language: English.

Excluded

1. Case reports, reviews, animal studies, and interventional randomized controlled trials.

2. Studies with a high proportion of participants with chronic hypertension, multiple pregnancies, fetal structural or chromosomal abnormalities.

3. Studies with unclear outcome definitions or unavailable key data.

4. Duplicate publications.

Intervention(s) or exposure(s) 1 change

Included

Exposure to fetal Doppler ultrasound assessments, including umbilical artery (UA), uterine artery (UtA), middle cerebral artery (MCA) Doppler indices, and cerebroplacental

ratio (CPR). Studies reporting any of these parameters for predicting adverse perinatal outcomes in patients with hypertensive disorders of pregnancy (HDP) are included.

Excluded
Studies without quantitative measurements of the above Doppler indices (e.g., only qualitative waveform descriptions or visual assessments without numerical values) are excluded. Studies focusing on other vessels (e.g., ductus venosus) without reporting the specified Doppler parameters, or those using Doppler for non-outcome prediction purposes in HDP patients, are also excluded. Abstracts, case reports, reviews, and studies without original primary data on the target Doppler indices are excluded.

Comparator(s) or control(s)
This review does not have any comparators

Study design 1 change
Only nonrandomized study types will be included.

Included
Observational cohort studies (prospective and retrospective designs) and prospective case-control studies that report fetal Doppler parameters and adverse perinatal outcomes in women with hypertensive disorders of pregnancy.

Excluded
Randomized controlled trials, case reports, review articles, editorials, letters, and studies with insufficient data or no clear diagnostic criteria for hypertensive disorders of pregnancy.

Context
Hypertensive disorders of pregnancy (HDP) are a leading cause of maternal and perinatal morbidity and mortality globally. Fetal Doppler ultrasound is a non-invasive tool used to assess fetal-placental circulation, with parameters including umbilical artery (UA), uterine artery (UtA), middle cerebral artery (MCA) Doppler indices, and cerebroplacental ratio (CPR). While these parameters are widely used in clinical practice, their predictive value for adverse perinatal outcomes (e.g., fetal growth restriction, small for gestational age, NICU admission, perinatal mortality) in HDP remains inconsistent across individual studies. This systematic review aims to synthesize the available evidence to quantify the diagnostic accuracy of these Doppler parameters in predicting adverse outcomes in women with HDP.

TIMELINE OF THE REVIEW

Date of first submission to PROSPERO
04 March 2026

Review timeline
Start date: 1 March 2026. End date: 10 April 2026.

Date of registration in PROSPERO
04 March 2026

AVAILABILITY OF FULL PROTOCOL

Availability of full protocol

A full protocol has been written but is not available because:

The full protocol is currently in preparation for submission to a peer-reviewed journal and will be made public upon publication.

SEARCHING AND SCREENING

Search for unpublished studies

Only published studies will be sought.

Main sources that will be searched 1 change

The main sources to be searched are *CENTRAL - Cochrane Central Register of Controlled Trials*, *Embase*, *PubMed* and *SCIE - Science Citation Index Expanded*.

Search language restrictions

There are no language restrictions.

Search date restrictions

Databases will be searched for articles published from 1 January 2016 and before by 4 March 2026.

Other methods of identifying studies

Other studies will be identified by: *contacting authors or experts*, *looking through all the articles that cite the papers included in the review ("snowballing" or forward citation searching)*, *reference list checking (backward citation searching)*, *searching conference proceedings*, *searching dissertation and thesis databases* and *searching trial or study registers*.

Link to search strategy

A full search strategy is available in the full protocol as described in the *Availability of full protocol* section

Selection process

Studies will be screened independently by at least two people (or person/machine combination) with a process to resolve differences.

Other relevant information about searching and screening

Two reviewers will independently screen titles and abstracts, then full texts. Discrepancies will be resolved by consensus or a third reviewer. Reference lists of included studies will be hand-searched for additional relevant studies. No language or publication date restrictions will be applied.

DATA COLLECTION PROCESS

Data extraction from published articles and reports

Data will be extracted independently by at least two people (or person/machine combination) with a process to resolve differences.

Authors will be asked to provide any required data not available in published reports.

Study risk of bias or quality assessment 1 change
Risk of bias will be assessed using: *Newcastle-Ottawa*

Data will be assessed independently by at least two people (or person/machine combination) with a process to resolve differences.

Additional information will be sought from study investigators if required information is unclear or unavailable in the study publications/reports.

Reporting bias assessment 1 change
Funnel plot and Egger's test will be used to assess publication bias.

Certainty assessment 1 change
The certainty of evidence will be assessed using the GRADE (Grading of Recommendations Assessment, Development and Evaluation) approach, considering risk of bias, inconsistency, indirectness, imprecision, and publication bias.

OUTCOMES TO BE ANALYSED

Main outcomes 1 change
The primary outcomes are the predictive performance indices of fetal Doppler parameters (UA, UtA, MCA, CPR) for adverse perinatal outcomes in HDP patients, including relative risk (RR) and 95% confidence interval (CI). Subgroup analyses will be performed based on different Doppler parameters and adverse outcomes.

Additional outcomes 1 change
Additional outcomes include:

1. The incidence of specific adverse perinatal outcomes, such as fetal growth restriction (FGR), preterm birth, low birth weight, and neonatal intensive care unit (NICU) admission.

2. Subgroup analyses of relative risk (RR) based on gestational age at Doppler measurement, type of HDP, and parity.

3. Meta-regression analyses to explore the impact of study-level covariates (e.g., sample size, study design) on the predictive performance of Doppler parameters.

PLANNED DATA SYNTHESIS

Strategy for data synthesis 1 change
For the meta-analysis of relative risk (RR) data, we will use the random-effects model to synthesize pooled RR with 95% confidence intervals (CI). Heterogeneity will be assessed using the I^2 statistic and Cochran's Q test. A random-effects model will be employed if substantial heterogeneity exists (I^2 > 50% or P < 0.10); otherwise, a fixed-effects model will be used. All primary meta-analyses will be conducted using RevMan software.

Stata software will be used for additional analyses, including Egger's test, trim-and-fill analysis, and meta-regression to explore potential sources of heterogeneity.

CURRENT REVIEW STAGE

Stage of the review at this submission 1 change

Review stage	Started	Completed
Pilot work	✓	✓
Formal searching/study identification	✓	✓
Screening search results against inclusion criteria	✓	✓
Data extraction or receipt of IPD	✓	
Risk of bias/quality assessment		
Data synthesis		

Review status
The review is currently planned or ongoing.

Publication of review results
Results of the review will be published in English and Chinese.

REVIEW AFFILIATION, FUNDING AND PEER REVIEW

Review team members
Mr Peng Zhou (review guarantor and contact) ORCID: 0009-0000-8593-4141. Gansu University of Chinese Medicine. China.

No conflict of interest declared.

Mr Siyuan Li. ORCID: 0009-0007-2815-684X. Gansu University of Chinese Medicine. China.

No conflict of interest declared.

Named contact
Mr Peng Zhou (979916249@qq.com). ORCID: 0009-0000-8593-4141. Gansu University of Chinese Medicine. China.

Review affiliation
Gansu University of Chinese Medicine

Funding source

Grant number
25JRRA334

Additional non-commercial funding information
Gansu Provincial Natural Science Foundation

Peer review

The study protocol was peer-reviewed by the Gansu Provincial Natural Science Foundation of China during the grant application process. The funding application (Grant No. 25JRRA334) underwent rigorous external expert evaluation and scientific review before being approved for funding.

ADDITIONAL INFORMATION

Additional information

This systematic review and meta-analysis is part of the research project "Application of prenatal ultrasound combined with chromosome microarray and whole-exome sequencing in fetal cardiac structural abnormalities" (Grant No. 25JRRA334) funded by the Gansu Provincial Natural Science Foundation of China. The study focuses on the predictive value of fetal Doppler parameters for adverse perinatal outcomes in hypertensive disorders of pregnancy.

Review conflict of interest

Declared individual interests are recorded under team member details.. No additional interests are recorded for this review.

Medical Subject Headings

Hypertension, Pregnancy-Induced; Ultrasonography, Doppler; Middle Cerebral Artery; Umbilical Arteries; Uterine Artery; Pregnancy Outcome

Revision note 1 change

This is the first revision of the initial registration. Changes have been made to align the methods section more closely with the study protocol described in the manuscript, including clarifying the main outcomes and analysis plan.

SIMILAR REVIEWS

Check for similar records already in PROSPERO 1 change

PROSPERO identified a number of existing PROSPERO records that were similar to this one (last check made on 5 May 2026). These are shown below along with the reasons given by that the review team for the reviews being different and/or proceeding.

- Diagnostic value of umbilical artery, middle cerebral artery, and cerebroplacental ratio Doppler for adverse perinatal outcomes in pregnant women with gestational hypertension: A systematic review and meta-analysis [published 24 September 2024] [CRD42024590263]. The review was judged **not to be similar**
- THE UTILITY OF CEREBROPLACENTAL RATIO (CPR) AND UTERINE ARTERY DOPPLER IN PREDICTING ADVERSE PERINATAL OUTCOMES IN LATE-ONSET FETAL GROWTH RESTRICTION: A SYSTEMATIC REVIEW AND META-ANALYSIS [published 27 January 2025] [CRD42025638977]. The review was judged **not to be similar**
- The Doppler In Pregnancy (DIP) study. A systematic review and meta-analysis to identify optimal artery Doppler studies for a prompt diagnosis of growth-restricted fetuses and adverse perinatal outcomes [published 25 September 2019] [CRD42019150551]. The review was judged **not to be similar**

PROSPERO version history 1 change

- Version 2.0, published 24 May 2026
- Version 1.0, published 04 Mar 2026

Disclaimer

The content of this record displays the information provided by the review team. PROSPERO does not peer review registration records or endorse their content.

PROSPERO accepts and posts the information provided in good faith; responsibility for record content rests with the review team. The guarantor for this record has affirmed that the information provided is truthful and that they understand that deliberate provision of inaccurate information may be construed as scientific misconduct.

PROSPERO does not accept any liability for the content provided in this record or for its use. Readers use the information provided in this record at their own risk.

Any enquiries about the record should be referred to the named review contact

Detailed Search Strategies for Electronic Databases *(Date of search: March 4, 2026)*

The search strategy was initially developed for the PubMed database using a combination of Medical Subject Headings (MeSH) and free-text terms. For the other electronic databases (Cochrane CENTRAL, Embase, and Web of Science), this core strategy was adapted strictly to suit their specific syntax requirements and thesaurus systems (e.g., Emtree for Embase).

1. **Search Strategy for PubMed**

#1 Population: ("Pre-Eclampsia"[Mesh] OR "Hypertension, Pregnancy-Induced"[Mesh] OR "Eclampsia"[Mesh]) OR ("Hypertension, Pregnancy Induced" OR "Pregnancy-Induced Hypertension" OR "Gestational Hypertension" OR "Pregnancy Induced Hypertension" OR "Pregnancy Transient Hypertension" OR "Pre Eclampsia" OR "Preeclampsia" OR "Pregnancy Toxemia*" OR "Edema-Proteinuria-Hypertension Gestosis" OR "Edema Proteinuria Hypertension Gestosis" OR "Hypertension-Edema-Proteinuria Gestosis*" OR "Proteinuria-Edema-Hypertension Gestosis" OR "Proteinuria Edema Hypertension Gestosis" OR "EPH Complex" OR "EPH Toxemia*" OR "EPH Gestosis" OR "Toxemia Of Pregnancy" OR "Toxemia Of Pregnancies" OR "Preeclampsia Eclampsia 1*" OR "Eclampsias")

#2 Exposure: ("Ultrasonography, Prenatal"[Mesh] OR "Ultrasonography, Doppler"[Mesh] OR "Umbilical Arteries"[Mesh] OR "Uterine Artery"[Mesh] OR "Middle Cerebral Artery"[Mesh] OR "Ductus Venosus"[Mesh]) OR ("Prenatal Ultrasonic Diagnoses" OR "Ultrasonic Prenatal Diagnoses" OR "Prenatal Ultrasonic Diagnosis" OR "Prenatal Ultrasonography" OR "Ultrasonic Prenatal Diagnosis" OR "Fetal Ultrasonography" OR "Doppler Ultrasound" OR "Doppler Ultrasounds" OR "Doppler Ultrasonography" OR "Doppler Ultrasound Imaging" OR "Doppler Ultrasound Imagings" OR "Umbilical Arteries" OR "Uterine Arteries" OR "Middle Cerebral Arteries" OR "ducts of Cuvier" OR "vena cardinalis" OR "cardinal vein" OR "common cardinal veins" OR "vena cardinalis communis" OR "common cardinal vein" OR "supracardinal veins" OR "subcardinal veins")

#3 Outcome: ("Pregnancy Outcome"[Mesh] OR "Fetal Growth Retardation"[Mesh] OR "Infant, Small for Gestational Age"[Mesh] OR "Infant, Low Birth Weight"[Mesh] OR "Fetal Distress"[Mesh] OR "Cesarean Section"[Mesh] OR "Intensive Care, Neonatal"[Mesh] OR "Perinatal Death"[Mesh]) OR ("Fetal Deaths" OR "Fetal Demise" OR "Fetal Mummification" OR "Intrauterine Growth Restriction" OR "Fetal Growth Restriction" OR "Intrauterine Growth Retardation" OR "Low-Birth-Weight Infant" OR "Low Birth Weight Infant" OR "Low-Birth-Weight Infants" OR "Low Birth Weight" OR

"Low Birth Weights" OR "Cesarean Sections" OR "Abdominal Delivery" OR "Caesarean Section" OR "Caesarean Sections" OR "Abdominal Deliveries" OR "Postcesarean Section" OR "Neonatal Intensive Care" OR "Perinatal Deaths" OR "Neonatal Death" OR "Neonatal Deaths")

#4 Final Combined Query: #1 AND #2 AND #3

2. **Search Strategy for Embase (Ovid/Embase.com syntax)** *Note: MeSH terms adapted to Emtree explosion (/exp) and free-text formatted for Embase.*

   #1 Population: ('pre-eclampsia'/exp OR 'pregnancy-induced hypertension'/exp OR 'eclampsia'/exp) OR ('Hypertension, Pregnancy Induced' OR 'Pregnancy-Induced Hypertension' OR 'Gestational Hypertension' OR 'Pregnancy Induced Hypertension' OR 'Pregnancy Transient Hypertension' OR 'Pre Eclampsia' OR 'Preeclampsia' OR 'Pregnancy Toxemia*' OR 'Edema-Proteinuria-Hypertension Gestosis' OR 'Edema Proteinuria Hypertension Gestosis' OR 'Hypertension-Edema-Proteinuria Gestosis*' OR 'Proteinuria-Edema-Hypertension Gestosis' OR 'Proteinuria Edema Hypertension Gestosis' OR 'EPH Complex' OR 'EPH Toxemia*' OR 'EPH Gestosis' OR 'Toxemia Of Pregnancy' OR 'Toxemia Of Pregnancies' OR 'Preeclampsia Eclampsia 1*' OR 'Eclampsias')

   #2 Exposure: ('prenatal ultrasonography'/exp OR 'doppler ultrasonography'/exp OR 'umbilical artery'/exp OR 'uterine artery'/exp OR 'middle cerebral artery'/exp OR 'ductus venosus'/exp) OR ('Prenatal Ultrasonic Diagnoses' OR 'Ultrasonic Prenatal Diagnoses' OR 'Prenatal Ultrasonic Diagnosis' OR 'Prenatal Ultrasonography' OR 'Ultrasonic Prenatal Diagnosis' OR 'Fetal Ultrasonography' OR 'Doppler Ultrasound' OR 'Doppler Ultrasounds' OR 'Doppler Ultrasonography' OR 'Doppler Ultrasound Imaging' OR 'Doppler Ultrasound Imagings' OR 'Umbilical Arteries' OR 'Uterine Arteries' OR 'Middle Cerebral Arteries' OR 'ducts of Cuvier' OR 'vena cardinalis' OR 'cardinal vein' OR 'common cardinal veins' OR 'vena cardinalis communis' OR 'common cardinal vein' OR 'supracardinal veins' OR 'subcardinal veins')

   #3 Outcome: ('pregnancy outcome'/exp OR 'intrauterine growth retardation'/exp OR 'small for date infant'/exp OR 'low birth weight'/exp OR 'fetal distress'/exp OR 'cesarean section'/exp OR 'neonatal intensive care'/exp OR 'perinatal mortality'/exp) OR ('Fetal Deaths' OR 'Fetal Demise' OR 'Fetal Mummification' OR 'Intrauterine Growth Restriction' OR 'Fetal Growth Restriction' OR 'Intrauterine Growth Retardation' OR 'Low-Birth-Weight Infant' OR 'Low Birth Weight Infant' OR 'Low-Birth-Weight Infants' OR 'Low Birth Weight' OR 'Low Birth Weights' OR 'Cesarean Sections' OR 'Abdominal Delivery' OR 'Caesarean Section' OR 'Caesarean Sections' OR 'Abdominal Deliveries' OR 'Postcesarean Section' OR 'Neonatal

Intensive Care' OR 'Perinatal Deaths' OR 'Neonatal Death' OR 'Neonatal Deaths')

#4 Final Combined Query: #1 AND #2 AND #3

3. **Search Strategy for Cochrane CENTRAL (Wiley Interface)** *Note: MeSH terms adapted to Cochrane [mh] syntax.*

   #1 Population: ([mh "Pre-Eclampsia"] OR [mh "Hypertension, Pregnancy-Induced"] OR [mh "Eclampsia"]) OR ("Hypertension, Pregnancy Induced" OR "Pregnancy-Induced Hypertension" OR "Gestational Hypertension" OR "Pregnancy Induced Hypertension" OR "Pregnancy Transient Hypertension" OR "Pre Eclampsia" OR "Preeclampsia" OR "Pregnancy Toxemia*" OR "Edema-Proteinuria-Hypertension Gestosis" OR "Edema Proteinuria Hypertension Gestosis" OR "Hypertension-Edema-Proteinuria Gestosis*" OR "Proteinuria-Edema-Hypertension Gestosis" OR "Proteinuria Edema Hypertension Gestosis" OR "EPH Complex" OR "EPH Toxemia*" OR "EPH Gestosis" OR "Toxemia Of Pregnancy" OR "Toxemia Of Pregnancies" OR "Preeclampsia Eclampsia 1*" OR "Eclampsias")

   #2 Exposure: ([mh "Ultrasonography, Prenatal"] OR [mh "Ultrasonography, Doppler"] OR [mh "Umbilical Arteries"] OR [mh "Uterine Artery"] OR [mh "Middle Cerebral Artery"] OR [mh "Ductus Venosus"]) OR ("Prenatal Ultrasonic Diagnoses" OR "Ultrasonic Prenatal Diagnoses" OR "Prenatal Ultrasonic Diagnosis" OR "Prenatal Ultrasonography" OR "Ultrasonic Prenatal Diagnosis" OR "Fetal Ultrasonography" OR "Doppler Ultrasound" OR "Doppler Ultrasounds" OR "Doppler Ultrasonography" OR "Doppler Ultrasound Imaging" OR "Doppler Ultrasound Imagings" OR "Umbilical Arteries" OR "Uterine Arteries" OR "Middle Cerebral Arteries" OR "ducts of Cuvier" OR "vena cardinalis" OR "cardinal vein" OR "common cardinal veins" OR "vena cardinalis communis" OR "common cardinal vein" OR "supracardinal veins" OR "subcardinal veins")

   #3 Outcome: ([mh "Pregnancy Outcome"] OR [mh "Fetal Growth Retardation"] OR [mh "Infant, Small for Gestational Age"] OR [mh "Infant, Low Birth Weight"] OR [mh "Fetal Distress"] OR [mh "Cesarean Section"] OR [mh "Intensive Care, Neonatal"] OR [mh "Perinatal Death"]) OR ("Fetal Deaths" OR "Fetal Demise" OR "Fetal Mummification" OR "Intrauterine Growth Restriction" OR "Fetal Growth Restriction" OR "Intrauterine Growth Retardation" OR "Low-Birth-Weight Infant" OR "Low Birth Weight Infant" OR "Low-Birth-Weight Infants" OR "Low Birth Weight" OR "Low Birth Weights" OR "Cesarean Sections" OR "Abdominal Delivery" OR "Caesarean Section" OR "Caesarean Sections" OR "Abdominal Deliveries" OR "Postcesarean Section" OR "Neonatal Intensive Care" OR "Perinatal Deaths" OR "Neonatal Death" OR "Neonatal Deaths")

#4 Final Combined Query: #1 AND #2 AND #3

4. **Search Strategy for Web of Science (SCIE)** *Note: Web of Science does not use MeSH terms. The MeSH concepts were combined with free-text terms into Topic Search (TS=) queries.*

#1 Population: TS=("Pre-Eclampsia" OR "Hypertension, Pregnancy-Induced" OR "Eclampsia" OR "Hypertension, Pregnancy Induced" OR "Pregnancy-Induced Hypertension" OR "Gestational Hypertension" OR "Pregnancy Induced Hypertension" OR "Pregnancy Transient Hypertension" OR "Pre Eclampsia" OR "Preeclampsia" OR "Pregnancy Toxemia*" OR "Edema-Proteinuria-Hypertension Gestosis" OR "Edema Proteinuria Hypertension Gestosis" OR "Hypertension-Edema-Proteinuria Gestosis*" OR "Proteinuria-Edema-Hypertension Gestosis" OR "Proteinuria Edema Hypertension Gestosis" OR "EPH Complex" OR "EPH Toxemia*" OR "EPH Gestosis" OR "Toxemia Of Pregnancy" OR "Toxemia Of Pregnancies" OR "Preeclampsia Eclampsia 1*" OR "Eclampsias")

#2 Exposure: TS=("Ultrasonography, Prenatal" OR "Ultrasonography, Doppler" OR "Umbilical Arteries" OR "Uterine Artery" OR "Middle Cerebral Artery" OR "Ductus Venosus" OR "Prenatal Ultrasonic Diagnoses" OR "Ultrasonic Prenatal Diagnoses" OR "Prenatal Ultrasonic Diagnosis" OR "Prenatal Ultrasonography" OR "Ultrasonic Prenatal Diagnosis" OR "Fetal Ultrasonography" OR "Doppler Ultrasound" OR "Doppler Ultrasounds" OR "Doppler Ultrasonography" OR "Doppler Ultrasound Imaging" OR "Doppler Ultrasound Imagings" OR "Umbilical Arteries" OR "Uterine Arteries" OR "Middle Cerebral Arteries" OR "ducts of Cuvier" OR "vena cardinalis" OR "cardinal vein" OR "common cardinal veins" OR "vena cardinalis communis" OR "common cardinal vein" OR "supracardinal veins" OR "subcardinal veins")

#3 Outcome: TS=("Pregnancy Outcome" OR "Fetal Growth Retardation" OR "Infant, Small for Gestational Age" OR "Infant, Low Birth Weight" OR "Fetal Distress" OR "Cesarean Section" OR "Intensive Care, Neonatal" OR "Perinatal Death" OR "Fetal Deaths" OR "Fetal Demise" OR "Fetal Mummification" OR "Intrauterine Growth Restriction" OR "Fetal Growth Restriction" OR "Intrauterine Growth Retardation" OR "Low-Birth-Weight Infant" OR "Low Birth Weight Infant" OR "Low-Birth-Weight Infants" OR "Low Birth Weight" OR "Low Birth Weights" OR "Cesarean Sections" OR "Abdominal Delivery" OR "Caesarean Section" OR "Caesarean Sections" OR "Abdominal Deliveries" OR "Postcesarean Section" OR "Neonatal Intensive Care" OR "Perinatal Deaths" OR "Neonatal Death" OR "Neonatal Deaths")

#4 Final Combined Query: #1 AND #2 AND #3

List of Excluded Studies at the Full-text Screening Stage and Reasons for Exclusion (n=10)

First Author (Year)	Title of the Study	Reason for Exclusion
Lin, X (2023)	Evaluation of Doppler indices (MCA & UA) and fetal outcomes: a retrospective case-control study in women with hypertensive disorders of pregnancy	**Insufficient data:** Core effect size data missing, unable to extract case numbers or calculate RR and 95%CI.
Ratiu (2019)	Doppler Indices and Notching Assessment of Uterine Artery Between the 19th and 22nd Week of Pregnancy in the Prediction of Pregnancy Outcome	**Insufficient data:** Core effect size data missing, unable to extract case numbers or calculate RR and 95%CI.
Yusuf (2017)	Uterine artery doppler velocimetry for the prediction of preeclampsia among high-risk pregnancies in low-resource setting...	**Insufficient data:** Core effect size data missing, unable to extract case numbers or calculate RR and 95%CI.
Chaves, M.T.P. (2016)	Ophthalmic artery dopplervelocimetry and pregnancy outcomes in preeclampsia	**Insufficient data:** Core effect size data missing, unable to extract case numbers or calculate RR and 95%CI.
Kienast, C. (2016)	Predictive value of angiogenic factors, clinical risk factors and uterine artery Doppler for pre-eclampsia and fetal growth restriction...	**Insufficient data:** Core effect size data missing, unable to extract case numbers or calculate RR and 95%CI.
Meroni (2023)	Is mid-gestational uterine artery Doppler still useful in a setting with routine first-trimester pre-eclampsia screening? A cohort study	**Inconsistent outcome definitions:** No core adverse perinatal outcomes (FGR, preterm birth, low birth weight) included.
Kumar (2025)	Serial evaluation of morphological and biophysical placental attributes in women with normal outcome and	**Inconsistent outcome definitions:** No core adverse perinatal outcomes

List of Excluded Studies at the Full-text Screening Stage and Reasons for Exclusion (n=10)

First Author (Year)	Title of the Study	Reason for Exclusion
	preeclampsia	(FGR, preterm birth, low birth weight) included.
Tokalioglu (2024)	Umbilical artery half peak systolic velocity deceleration time: a novel Doppler parameter for prediction of neonatal outcomes in pregnant women with preeclampsia	**Inconsistent outcome definitions:** No core adverse perinatal outcomes (FGR, preterm birth, low birth weight) included.
Babic (2020)	Intraplacental Villous Artery Doppler as an Independent Predictor for Placenta-Mediated Disease and Its Comparison with Uterine Artery Doppler...	**Unclear diagnostic criteria:** HDP diagnosis not in line with international standards, ambiguous study population enrollment.
Jie, M (2023)	Second trimester abnormal uterine artery Dopplers and adverse obstetric and neonatal outcomes when PAPP-a is normal	**Unclear diagnostic criteria:** HDP diagnosis not in line with international standards, ambiguous study population enrollment.
